# A systematic review of approaches to improve practice, detection and treatment of unhealthy alcohol use in primary health care: a role for continuous quality improvement

**DOI:** 10.1186/s12875-020-1101-x

**Published:** 2020-02-13

**Authors:** Monika Dzidowska, K. S. Kylie Lee, Claire Wylie, Jodie Bailie, Nikki Percival, James H. Conigrave, Noel Hayman, Katherine M. Conigrave

**Affiliations:** 1 Faculty of Medicine and Health, Discipline of Addiction Medicine, NHMRC Centre of Research Excellence in Indigenous Health and Alcohol, The University of Sydney, Lev 6, King George V Building (C39), The University of Sydney, NSW 2006 Australia; 2 Centre for Alcohol Policy Research, La Trobe University, Level 5, HS2, Bundoora, VIC 3086 Australia; 3 Faculty of Medicine and Health, Translational Australian Clinical Toxicology Program, The University of Sydney, Lev3, 1-3 Ross Street (K06), The University of Sydney, NSW 2006 Australia; 4 The University of Sydney, Faculty of Medicine and Health, University Centre for Rural Health, 61 Uralba Street, Lismore, NSW 2480 Australia; 5 Faculty of Health, Australian Centre for Public and Population Health Research, University of Technology Sydney, UTS Building 10, 235-253 Jones Street, Ultimo, NSW 2007 Australia; 6 Southern Queensland Centre of Excellence in Aboriginal and Torres Strait Islander Primary Health Care (Inala Indigenous Health Service), 37 Wirraway Parade, Inala, QLD 4077 Australia; 7 School of Medicine, Griffith University, Griffith Health Centre (G40), Gold Coast campus, Gold Coast, QLD 4222 Australia; 8 School of Medicine, University of Queensland, Herston Road, Herston, QLD 4006 Australia; 9 Sydney Local Health District, Royal Prince Alfred Hospital, Drug Health Service, King George V Building, 83-117 Missenden Road, Camperdown, NSW 2050 Australia

**Keywords:** Alcohol, Unhealthy alcohol use, Alcohol use disorders, Implementation, Primary health care, Continuous quality improvement, Screening, Treatment, Brief intervention

## Abstract

**Background:**

Unhealthy alcohol use involves a spectrum from hazardous use (exceeding guidelines but no harms) through to alcohol dependence. Evidence-based management of unhealthy alcohol use in primary health care has been recommended since 1979. However, sustained and systematic implementation has proven challenging. The Continuing Quality Improvement (CQI) process is designed to enable services to detect barriers, then devise and implement changes, resulting in service improvements.

**Methods:**

We conducted a systematic review of literature reporting on strategies to improve implementation of screening and interventions for unhealthy alcohol use in primary care (MEDLINE EMBASE, PsycINFO, CINAHL, the Australian Indigenous Health InfoNet). Additional inclusion criteria were: (1) pragmatic setting; (2) reporting original data; (3) quantitative outcomes related to provision of service or change in practice. We investigate the extent to which the three essential elements of CQI are being used (data-guided activities, considering local conditions; iterative development). We compare characteristics of programs that include these three elements with those that do not. We describe the types, organizational levels (e.g. health service, practice, clinician), duration of strategies, and their outcomes.

**Results:**

Fifty-six papers representing 45 projects were included. Of these, 24 papers were randomized controlled trials, 12 controlled studies and 20 before/after and other designs. Most reported on strategies for improving implementation of screening and brief intervention. Only six addressed relapse prevention pharmacotherapies. Only five reported on patient outcomes and none showed significant improvement. The three essential CQI elements were clearly identifiable in 12 reports. More studies with three essential CQI elements had implementation and follow-up durations above the median; utilised multifaceted designs; targeted both practice and health system levels; improved screening and brief intervention than studies without the CQI elements.

**Conclusion:**

Utilizing CQI methods in implementation research would appear to be well-suited to drive improvements in service delivery for unhealthy alcohol use. However, the body of literature describing such studies is still small. More well-designed research, including hybrid studies of both implementation and patient outcomes, will be needed to draw clearer conclusions on the optimal approach for implementing screening and treatment for unhealthy alcohol use. (PROSPERO registration ID: CRD42018110475).

## Background

Unhealthy alcohol use involves a broad spectrum of conditions from hazardous or risky drinking to the diagnosis of alcohol use disorder. ICD-11 defines hazardous drinking as use that increases the risk of harmful physical or mental health consequences to the user or to others, while disorders due to alcohol involve use patterns that have already caused harm or dependence [1]. Evidence-based management of unhealthy alcohol use in primary health care (PHC), particularly the use of screening and brief intervention (SBI), has been advocated since the World Health Organization (WHO) called for the development of strategies and guidelines for SBI applicable in PHC settings [2–5]. SBI is now widely accepted as best practice and recommended by both national and international guidelines [6].

Meta-analyses of studies of implementation of alcohol screening and treatment have shown that multi-faceted programs with longer duration and alcohol-focused programs are better at achieving improvements. Specifically, programs oriented towards multiple-organizational levels, as well as studies longer than 12 months were associated with significant effects on improvement of implementation of screening and/or brief intervention compared to single strategy programs [7, 8]. Programs combining strategies that targeted the clinician, organization and patient were more effective in decreasing alcohol consumption than clinician-only strategies [8]. However, sustained and systematic implementation of evidence-based care for alcohol use in PHC continues to be a problem [6, 9–12]. Furthermore, there is little evidence of significant effects of implementation strategies on patients’ alcohol consumption [8]. Barriers, such as time pressures, staff retention, lack of training and leadership, as well as the clinicians’ perception of alcohol discussions as sensitive, have been identified [6, 11, 13]. To improve detection and treatment of unhealthy alcohol use, more work is needed to develop and test approaches that are sensitive to facilitators and barriers in an individual PHC setting.

Continuous quality improvement (CQI) in health care has been defined as “*a structured organizational process for involving people in planning and executing a continuous flow of improvement to provide quality health care that meets or exceeds expectations*” [14]. Originating from industrial process improvement approaches, this approach has been used in health care since the 1990s [14, 15]. CQI is designed to improve health care by using data to identify where services are doing well and not so well, implementing and monitoring corrective action and then reviewing its effectiveness, in continuous improvement cycles. Studies, including the largest CQI program in Australia [16], have shown that with consistent policy and infrastructure it can facilitate ongoing improvement of PHC service delivery and subsequently, better health outcomes [16–19]. This largest program includes research in Aboriginal community controlled primary health care services [16]. However, to our knowledge, there is no literature review specifically on the use of CQI strategies in improving service provision for unhealthy alcohol use in the PHC setting.

This systematic review aims to: 1) describe types, levels and duration of implementation strategies to improve screening and treatment for unhealthy alcohol use in PHC, and their outcomes, as available in peer-reviewed literature; 2) investigate to what extent elements of CQI are being used in these strategies; 3) compare characteristics of programs with all CQI elements with programs that do not have these elements.

## Methods

We performed a systematic review of peer-reviewed literature from January 1990 to September 2018 (referred to from here on as ‘reports’). The year 1990 was chosen because it marked the beginning of the decade following the WHO’s first release of guidelines for alcohol screening and brief intervention, as well as the beginnings of CQI in health care [3, 14, 15].

### Search strategy

To construct the search strategy, we first conducted a broad text-word search in MEDLINE.

From this search (14,764 results) we identified a set of representative reports that met the inclusion criteria (a sentinel set; *n* = 25) by systematically screening 20% of the search results for abstracts that met the inclusion criteria. Medical Subject Headings (MeSH) and keywords of the 25 sentinel articles were then used to progressively refine the search strategy: subject headings and subheadings not already in the original search strategy were identified and used to modify the search strategy. Retention of the sentinel set was checked with each modification. The strategy was then further refined through an independent review by an expert in drug and alcohol health services research. The resulting final strategy consisted of three groups of search terms reflecting the problem (e.g. alcohol, binge drinking), setting (e.g. primary care, general practice), and intervention (e.g. program. strategy) of interest to this review. A summary of the strategy is presented in Table 1. This strategy was applied to MEDLINE, EMBASE and PsycINFO with modifications made as required. An adapted set of search terms was used in CINAHL and the Australian Indigenous Health InfoNet. Search results were restricted to English language. Hand searches were performed on reference lists of 21 major reviews, sourced from Cochrane (including Cochrane EPOC and Cochrane Drugs and Alcohol Review Group) and the above literature search. The final set included for analysis was checked for any additional reports. A detailed protocol and search strategy are available in the international prospective register of systematic reviews, PROSPERO (ID CRD42018110475), https://www.crd.york.ac.uk/prospero/.

**Table 1:** Summary of the final search strategy (MEDLINE)

Search term group (number of search terms^a^ entered)	Examples of search terms
Implementation strategies and treatments (38 terms)	Mass Screening; Counseling; Evaluation Studies as Topic; Delivery of Healthcare; Total Quality Management; PDSA; Pharmacotherapy.mp; Health Check*.mp; organi* interv*.mp
Alcohol drinking (5 terms)	Alcohol*mp; Alcoholism; Binge Drinking; Alcohol Drinking; Alcoholic Intoxication
Primary Health Care (7 terms)	Primary Health Care; Preventative Health Services; commun$ health.mp; Physicians, Family; Physicians, Primary Care; Family Practice; General Practice

^a^Number of search terms entered represents the number of unexpanded MeSH subject headings and text key words entered into MEDLINE search. All MeSH subject headings were expanded

Reports were included if they described experimental or observational studies that: (1) were conducted in a pragmatic PHC setting, that is the strategies were integrated into routine practice and delivered primarily by existing PHC staff [20]; (2) described an intervention/initiative/program designed to improve service provision or improve evidence-based practice to address unhealthy alcohol use; (3) reported original data; and (4) reported quantitative outcomes related to provision of service or change in practice for unhealthy alcohol use. Reports that utilized clinician self-reported outcome measures were included only if they quantified the change in service provision. Exclusion criteria comprised non-original data reports, reviews, commentaries and editorials, method reports, a citation without abstract available, and conference abstracts.

### Data extraction

Literature searches were downloaded into Endnote X8.2 and duplicates removed. Irrelevant reports and ineligible publication types were removed at the stage of the title screen. Titles and abstracts of the resulting set were independently reviewed for inclusion criteria by two reviewers (MD, CW). Where agreement could not be reached a third reviewer (KC or KL) was consulted. Full text review was performed by MD and CW with further discrepancies discussed with KC. Data from the final set was extracted by MD in consultation with KC and KL.

We extracted the following data:

- information on study design and setting
- description of the improvement strategy including targeted clinical actions
- whether strategy was multifaceted (that is they employed more than one component [e.g. training plus financial incentive] to target implementation barriers and achieve improvement)
- organizational levels targeted by the strategy, defined as:
  - National – targeting the health care system for an entire population
  - Health system – targeting organizational structures within a health system (e.g. local, state-based, or private health insurance company)
  - Practice – targeting individual primary care practices
  - Clinician – targeting clinicians working within PHC practice settings
  - Patient – targeting the patient or population being served by the practices
- details of follow-up
- type of outcome measure and outcomes.

### Identifying CQI elements

Because in academic literature, CQI methodology is not always clearly identified [15, 21, 22], we screened for the presence of three essential CQI elements defined by Rubenstein et al. [22]:

(i) Using ‘systematic data guided activities’ to identify problems and achieve improvement
(ii) ‘designing with local conditions in mind’
(iii) using an ‘iterative development and testing process’

We defined element (i) as present if there was clear indication that the improvement strategy included systematic use of data to conduct assessment of the problem to be addressed and/or to diagnose improvement and a response to this data that modified the improvement strategy. We defined element (ii) as present if there was clear indication of designing and/or allowing adaptation of strategies to fit the special characteristics of the local setting. Element (iii) required evidence that the data collection and response in element 1 was conducted in at least two cycles. The elements were coded as ‘present’, ‘absent’ or ‘unclear’. For the purposes of descriptive analyses below any instances of ‘unclear’ were treated as absent.

Descriptive analysis was performed on all reports that met the selection criteria as well as on the subset of reports describing initiatives that included all three CQI elements.

## Results

**Fig. 1:**
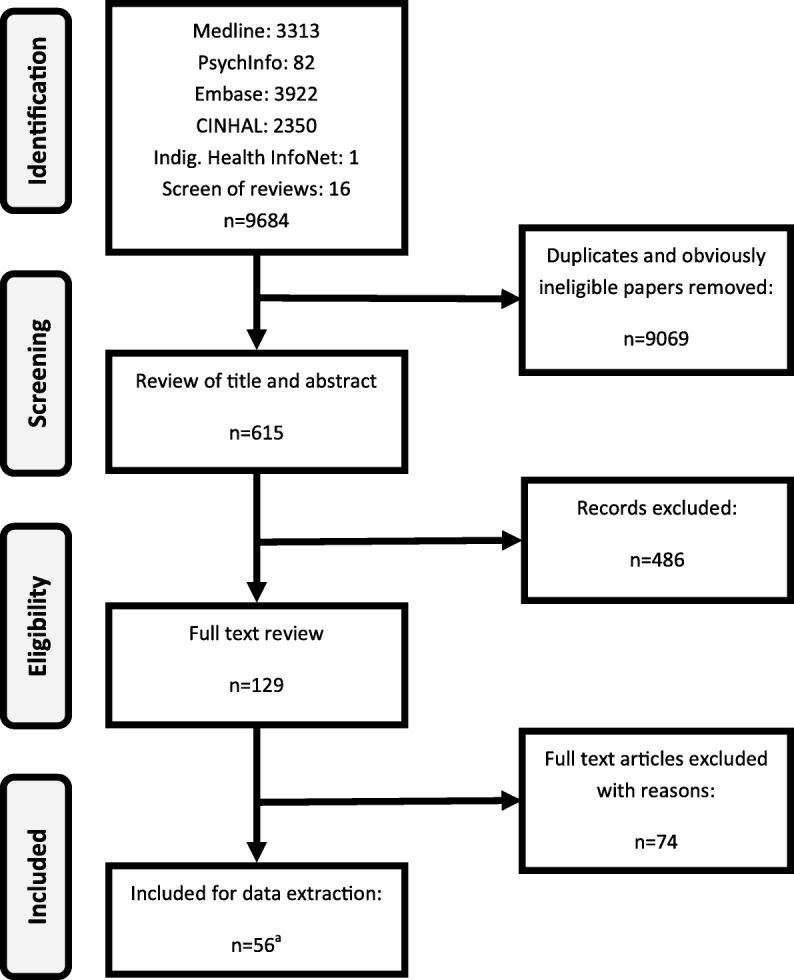
Search strategy flow chart. ^a^One additional paper was identified from final set of reports included in analysis

Fifty-six reports representing 45 studies were included in the review (Fig. 1). Of these, 24 reports were randomized controlled trials (RCTs) [23–46], 12 were controlled designs [47–58] and 20 were before/after and other designs [59–78]. Thirty-five were alcohol-specific, while 21 focused on broader prevention (Table 2).

**Table 2:** Characteristics of studies included in the review

Study (***n*** = 56)^**a**^	Sample size			Strategy	Targets			Main outcomes	CQI elements^**c**^		
	Patients	Clinicians	Sites		Clin.action^**b**^	Org. level	M/faceted		1	2	3
Randomized controlled trials											
WHO collaborative Project (Phase 3)											
Gomel 1998 AUS Alcohol [33]	94,481	Phase 1: 628 Phase 2: 161 (1 per site)	628 161	Phase 1: mailout (c); telemarketing; academic detailing. Phase 2: written guidance (c); training; training + min support; training + ongoing support	**S, BI**	3,4	Y	Higher uptake if academic detailing or telemarketing; higher screening in training or training + max support cf. other arms; advice significantly higher in max support arms	N	N	N
Hansen et al. 1999 DNK Alcohol [34]	na	143	na	Phase 1 only	**S,** BI	4	N	Higher uptake if academic detailing or telemarketing. No significant differences control cf. intervention arms	N	N	N
Kaner 1999 GBR Alcohol [39]	11,007	128	128	Phase 2 only (excluding the training + min support arm).	**S, BI**	4	Y	Increased implementation, screening and intervention in training + support.	N	N	N
Funk 2005 AUS, BEL, DNK, NZ, ESP, GBR Alcohol [32]	60,989	Phase 1: 3436, Phase 2: 727 (1 per site)	3436 727	Phase 1 & 2 (excluding the training + min support arm)	**S, BI**	3,4	Y	Increased uptake if academic detailing or telemarketing. Increased screening and advice giving if training or training + support.	N	N	N
Anderson 2004 AUS, BEL, ESP, GBR Alcohol [25]	na	Phase 1: 2924; Phase 2: 632 (1 per site)	2924 632	Phase 1 & 2 (excluding the training + min support arm)	**S, BI**	3,4	Y	Sub-analysis of Funk 2005: Increased screening and BI if physicians secure and committed in working with drinkers	N	N	N
ODHIN											
Anderson 2016 ESP, GBR, NLD, POL, SWE Alcohol [23]	Mean: 1500 consults/site (baseline)	746	120	Country guidelines summary (c); training and support (TS), Financial reimbursement (FR), access to referral to eBI and combinations of these	**S,** BI	3,4	Y	During 12-week implementation: Increased screening in TS arm and FR arm. Increased intervention (screening or advice) in TS, FR, TSFR and TSFReBI. No effect on giving advice to screen positive pts.	N	N	N
Bendtsen 2016 ESP, GBR, NLD, POL, SWE Alcohol [26]	As above	350	60	As above	S, **BI**	3,4	Y	No association between eBI and increase in screening. Increased proportion of screen-positive pts. given BI. Low pt. and provider uptake rates of eBI	N	N	N
Anderson 2017 ESP, GBR, NLD, POL, SWE Alcohol [24]	As above	746	120	As above	S**, BI**	3,4	Y	At 9 months follow-up: Increased intervention rates in TS. Reduction of intervention rates in all arms but reduction in TS arm was smaller.	N	N	N
CN SNAP											
Chan 2013 AUS Broad prev [28].	na	129	4	Training (5As); Integrating assessment/prompts into initial visits; referral directory; resources including. Guides for nurses, action plans for each risk factor.	**S,** BI, RT	3,4	Y	Increased self-reported screening at 6 and 12 months (validated scale). No effect on self-reported management or referral.	N	N	N
Harris 2013 AUS Broad prev [35].	804	na	4	As above.	BI, **RT**	3,4	Y	Increase in pt-reported referrals in intervention group at 3 months cf. baseline. No significant changes in self-reported alcohol consumption.	N	?	N
Other RCTs											
Bonevski 1999 AUS Broad prev [27].	2917	19	na	Computerized feedback system: guidelines, goal setting for GPs, GP feedback on performance in other health screening (not alcohol).	**na**	4	Y	Although not targeted by intervention, at 3-month follow-up classification of hazardous/harmful drinkers more accurate in intervention arm cf. controls.	N	N	N
Dubey 2006 CAN Broad prev [30].	1117	38	4	Gender based preventative checklist prompt with evidence-based recommendations.	**S**	4	N	Significant increase in alcohol history intake between baseline and follow-up in intervention arm. Increase significantly associated with intervention.	N	N	N
Chossis 2007 Switzerland Alcohol [29]	260	27	2	Training; summary checklist, textbook and pt. education materials.	**BI**	4	Y	Intervention residents conducted more components of BI: more likely to explain safe drinking limits provide feedback, seek pt. opinions on drinking limits, after training but not at follow-up; no effect on pt. drinking patterns.	N	N	N
Friedmann 2006 USA Alcohol [31]	164	18	2	Maintenance care training for alcohol problems in remission (5As); follow-up academic detailing; booster training; materials for pts. and clinicians; pt. record prompt (paper).	**S,** BI**, RP, RT**	4	Y	Intervention pts. more likely to report clinician took alcohol history. Intervention clinicians who took alcohol history more likely to assess prior and planned alcohol treatment, offer of prescriptions and referral.	N	N	N
Harris 2015 AUS Broad prev [36].	21,848	122	32	Training of practice staff and QI facilitators; audit and feedback; site visits with goal setting; pt. education and referral materials; implementation support; facilitator support.	**S**	3,4	Y	At 12 months follow-up increase in odds of alcohol recording of alcohol consumption in the intervention compared to control. No significant change in the level of risk factors based on audit data.	Y	Y	Y
Haskard 2008 USA Broad prev [37].	2196	156	3	Physician training; pt. training.	**BI**	4,5	Y	Significant upward trend in counselling to quit alcohol at time 6-months post training after initial expected drop at 1-month post training.	N	N	N
Kaner 2003 GBR Alcohol [38]	5541	na	212	Phase 1: mailout (c); telemarketing; academic detailing. Phase 2: written guidance (c); training; training + ongoing support (directed at nurses).	S, **BI**		Y	Increased implementation in training and training + support. Increased BI in training and training + support. Fewer pt. management errors in controls.	N	?	N
Krist 2016 USA Broad prev [40].	2913	156	18	MOHR: self-administered health behaviour questionnaire; MOHR summary and feedback for pts.; a summary of positive MOHR for clinicians; optional training for clinicians; freedom of method of implementation.	**S, BI,** RT	3,4	Y	Significantly higher screening for alcohol and goal setting to reduce risky drinking in intervention arm compared to control. No significant changes in referrals. No significant changes in alcohol consumption.	N	Y	N
Ornstein 2013 USA Alcohol [43]	26,005	77	20	Pre-intervention visit; electronic screening/intervention prompt and resource template; network meeting to discuss facilitators and barriers and to develop implementation plans; performance feedback; on-site support visits.	**S, BI, RP**	3,4	Y	Early Intervention (EI) phase: increased odds of screening and BI in EI cf. delayed intervention (DI); performance stable at DI phase. DI phase: increased odds of screening in DI pts. cf. EI phase. Increased prescription of AUD medication in EI pts. at DI phase.	Y	Y	Y
Mertens 2015 USA Alcohol [41]	420,946	554	54	Physician (PCP), Non-physician providers (NPP), Medical Aid (MA) arms. Training: PCP trained in all of SBIRT, MAs trained to ask screening question, NPPs trained to ask weekly drinking questions, AUD screener and BI and RT. All arms: Screening + automated prompts added to electronic health record; implementation support; audit + feedback.	**S, BI, RT**	3,4	Y	Higher screening rates in NPP, MA and PCP cf. controls. Higher BI and referrals in PCP cf. other arms (No difference between NPP, MA and controls).	N	Y	Y
Navarro 2012 AUS Alcohol [42]	155,170	na	20	Feedback letter to GP: prescription + community dependence rates, information on pharmacotherapies + behavioural interventions; recommendation to increase prescribing to reduce heavy alcohol consumption.	**RP**	4	N	Increased acamprosate but decreased naltrexone prescribing cf. controls.	N	N	N
Rose 2008 USA Alcohol [44]	27,591	na	22	NIAAA screening guidelines; instructions to develop/adapt screening template in electronic MRs; performance feedback and review; on-site visits with training, development of action plan; network meetings. Screening template (5As with AUDIT-C + question /diagnosis/recording prompts).	**S, BI, RT**	2,3,4	Y	Screening, counselling odds higher in intervention cf. controls. Improvements over time greater in intervention arm. Reduced PB in pts. given brief counselling or referral but No significant reduction for intervention cf. controls.	Y	Y	Y
Saitz 2003 USA Alcohol [45]	212	41	1	Clinical prompt: Results of CAGE assessment + recommendations attached to pt. record.	**BI,** RT	4	N	Faculty physicians in intervention arm more likely cf. controls to give advice, discuss associated problems. No significant difference in outcomes for residents in intervention cf. control arm. At 6-months, intervention arm pts. who saw residents had fewer drinks/drinking day but no between group differences.	N	N	N
van Beurden 2012 NLD Alcohol [46]	1502	124	82	3 components targeting: [1] professionals: training, guidelines, reminder cards [2]; organization: feedback report, facilitation of external specialist support, implementation support [3]; Pt-directed: letters, leaflets, self-help booklets, poster, personal feedback based on consumption.	S, BI	3,4,5	Y	No difference in improvement in screening or BI in intervention cf. control.	?	Y	N
Non randomized controlled studies											
Bradley 2002 USA Alcohol [47]	68	34	2	Clinical prompt: pt-specific positive screening result at each visit.	**BI**	4	N	Intervention group more likely to discuss alcohol use cf. controls.	N	N	N
Hamilton 2014 GBR Alcohol [48]	211,834	na	30	Pay-for-performance scheme (QOF+) to extend alcohol screening; computer templates; in-practice training.	**S, BI,** RT	2	Y	Increased screening in eligible and Non-eligible group cf. baseline. Eligible pts. more likely to receive ASBI and full AUDIT than Not eligible.	N	N	N
Harris 2017 USA Alcohol [49]	2952	199	3	3 components targeting [1] Local champion: training + support, monthly teleconferences, access to national champions, website, pt. dashboard [2]; Providers: training + support, website, access to local champions, pt. dashboard, reminder emails [3]; Pt education + activation: mailed materials.	**RP**	2,3,4,5	Y	Increased odds of filling a prescription during implementation in the three sites, however not significant at one site when stratified by site. No significant changes cf. matched controls.	N	?	N
Khadjesari 2017 GBR Alcohol [50]	261,424	na	na	Pay-for-performance scheme for specific clinical areas.	**S**	1,2	N	Increase in alcohol recording rate ratio over 13 years in case group cf. control group.	N	N	N
Mason 1997 GBR Alcohol [51]	1417	na	4	Nurse-counsellor providing counselling services to practices + training to physicians.	**S, BI,** RT	3	Y	Increase in: recording of consumption and identification of problem drinkers (all intervention sites), identification of pts. drinking above recommended limits and advice (2 sites). No increases in referrals.	N	N	N
McElwaine 2014 AUS Broad prev [52].	1989	570	17	Local leadership engagement, electronic MR modification, training, implementation support, audit + feedback.	S**, BI,** RT	2,3,4	Y	Increase in odds of provision of Brief Advice from baseline to follow-up in intervention cf. controls. No changes in screening or referrals.	Y	?	Y
O’Donnell 2016 GBR Alcohol [53]	106,700	99	16	Two pay-for-performance schemes: National (DES) - for each newly registered pt. screened; Local (LES) - for each new pt. over 16 positive for risky drinking + received BI.	**S, BI**	1,2	N	Rates of short screening (FAST or AUDIT-C) or AUDIT lowest in non-incentivised and highest in DES. Rates of alcohol intervention lowest in non-incentivised and highest in DES. Significance Not reported.	N	N	N
Onders 2014 USA Broad prev [54].	23,000 visits/year Indigenous	10	1 (cf national service)	Electronic clinical reminders (CR) using PDSA: [1] data-driven ID of need [2]; Pilot test CR [3]; Expand to all providers [4]. audit +feedback [5]; Delegation of CR to other staff.	**S**	3,4	Y	Increased screening from 35 to 70% cf. IHS (smaller increase 40–48%) cf. other IHS.	Y	Y	Y
Ozer 2005 USA Broad prev [55].	T_0_ = 226(i), 246(c); T_1_ = 551(i), 260(c); T_2_ = 940(i), 405(c) Adolescents	76	4	Clinician training; facilitated implementation of screening and charting forms tailored to local conditions. (Setting: paediatric PHC).	**S, BI**	3,4	Y	Increased screening and counselling post implementation both elements (intervention cf. controls). Increases associated with post training. No additional increases post tool implementation.	N	?	N
Thomas 2014 SWE Broad prev [56].	T1 = 888 T2 = 994	T1 = 120 T2 = 132	6	Implementing screening for risky behaviour + BI and referrals to in-house multidisciplinary team; compulsory components: multidisciplinary teams + managers, meetings, in-house referral workflows.	S, BI	3,4	Y	No difference in alcohol consumption discussion rates in intervention cf. control at 3 years. Significantly higher alcohol discussion rates in control cf. intervention at 5 years.	N	?	N
Wiggers 2017 AUS Broad prev [57].	5369	~ 1400	56	Policy + leadership engagement; modifying information systems; training; audit and feedback; implementation support, information and resources.	**S, BI,** RT	2,3,4	Y	Increased alcohol consumption assessment and advice cf. control (stepped wedge). No increases in referrals.	Y	Y	Y
Wilson 1992 GBR Broad prev [58].	4471	16	10	Increased consultation booking time from 6 to 10 min per pt.	**na**	3	N	Increased recording of alcohol education and in pt-reported discussion about alcohol.	N	N	N
Before/after and other designs											
Healthy Habits											
Seale 2005a USA Alcohol [77]	3387	35	1	Formation of lead committee (monitoring + recommendations); strategy development, modification of pt. record + workflows to include SBI; clinician training.	**S, BI,** RT	3,4	Y	Increased screening + intervention. Clinicians intervened more often when prompted with AUDITs. Periodic evaluation resulted in modifications, which resulted in progressive increases in screening.	Y	Y	Y
Seale 2005b USA Alcohol [76]	1052	38	1	Formation of leading committee (monitoring + recommendations); strategy development, modification of pt. record + workflows to include SBI; clinician training.	S, **BI**	4	Y	No significant differences in problem drinking (PD) identification before and after intervention. After training, greater increase in advice giving in residents cf. faculty.	Y	Y	Y
Johnson 2013 USA Alcohol [69]	288	na	1	SBI workflow additions to protocols implemented above: Single Alcohol Screening Question (SASQ); checkbox in pt. record for BI; booster training.	**S,** BI	3,4	Y	Screening rates using AUDIT-C plus SASQ exceeded 90% but no significant changes. Increased identification of UAU at 6 weeks and 6 months.	?	?	?
VA program											
Lapham 2012 USA Alcohol [70]	6788	na	na	Health system-wide incentives-linked performance measure (PM) + BI electronic clinical reminder (CR); freedom of adaptation but core PM components required; BI clinician training optional.	**BI**	2,3,4	Y	Recording of advice increased continuously from baseline year, after PM announcement, PM implementation, and CR dissemination.	N	Y	N
Chavez 2016 USA Alcohol [62]	225,912	na	na	As above.	**BI**	2,3,4	Y	Increased pt-reported advice to pts. with moderate-severe alcohol misuse from baseline with plateau in two final years.	N	Y	N
ABCD											
Si 2007 AUS Broad prev [78].	360 Indigenous	na	12	Single intervention cycle includes: initial systems audit + records audit; identify priorities + design improvement strategy; audit + feedback to monitor + identify new priorities.	BI	3	Y	Alcohol counselling/advice increased significantly at Year 2 audit.	Y	Y	Y
Gibson-Helm 2016 AUS Broad prev [65].	2220 (Indigenous Pregnant women)	na	50	As above.	S, BI	3,4	Y	Increased odds of screening and BI with each cycle. Evidence of a trend in increased number of CQI cycles and increase in BI.	Y	Y	Y
Other											
Aalto 2003 Finland Alcohol [59]	1449	24	2	Collaborative BI implementation development; training; implementation support; reminders in local and professional publications; distribution of AUDIT to all households.	S, BI	2,3,4,5	Y	No statistically significant differences.	N	?	N
Aspy 2008 USA Broad prev [60].	600	30	9	Audits + feedback; training; practice facilitation/support; meetings between participating practice teams; Nurses, medical assistants, trained in screening and very brief interventions (VBI) e.g. referrals and handouts. Clinicians trained in BI.	S, BI	2,3,4	Y	No significant changes in alcohol screening or VBI or BI cf. baseline. Pts less likely to screen positive for UAU at end of study cf. baseline. Screening increased if alcohol was the target in first two cycles. Addition of more than two target behaviours appeared to negatively impact previous targets.	Y	Y	Y
Bobb 2017 USA Alcohol [61]	53,133	na	3	3 strategies: [1] Enabling teams: recruitment + CQI training of site champions, development + implementation support, regular education on CQI, SBI + AUD treatment, information sharing between sites [2]. Support via electronic health record: screening, BI, AUD prompts [3]; Monitoring + feedback: PDSA; meetings.	**S,** BI, RP, RT	2,3,4	Y	Increased screening and assessment post-implementation cf. baseline. Effects sustained 1 year later + increased new AUD diagnoses. Increased treatment within 30- and 90-days post diagnosis (driven mainly by one site).	Y	Y	Y
Clifford 2013 AUS Alcohol [63]	9322	na	4	Training; treatment guidelines; electronic assessment tool; implementation support	**S, BI**	3,4	Y	Increased records of screening. Increased records of BI overall but Not significant in individual ACCHS.	N	Y	N
Cowan 1994 USA Alcohol [64]	910	11	1	Clinician training.	**Y** **S**	4	N	Significant increase in recording of drinking histories.	N	N	N
Gilkes 2017 AUS Broad prev [66].	2608	35	na	Clinical audit + feedback by medical students to GP supervisors.	**S**	4	N	Increased record of alcohol consumption.	N	N	Y
Gowin 2012 POL Broad prev [67].	1060	106	na	Regional training program.	**S**	2,4	N	Increased screening.	N	N	N
Holtrop 2009 USA Broad prev [68].	1965	na	20	Record audits + practice assessment; choice of improvement plan based on 5As, priority risk behaviour or both; support in planning + implementation; audit + feedback at end of study.	S, BI	3,4	Y	No practice chose alcohol as target. However, increased alcohol screening but non-significant when adjusted for clustering.	Y	Y	?
Lawner 1997 USA Alcohol [71]	297	15	1	Training of faculty members to give performance feedback to residents with a feedback form.	**S**	4	N	Increased record of alcohol consumption. Increased use of CAGE.	N	N	N
Lustig 2001 USA Broad prev [72].	532 (Adolescents)	63	3	Clinician training (Setting: paediatric PHC).	**S,** BI	4	N	Increased screening.	N	N	N
Marco-Garcia 1999 ESP Broad prev [73].	(1500–2000/doctor, 42 doctors)	84	3	Formation of task force; collaborative program development; consensus on indicators + evaluation criteria; regular audit; action in response to audit.	**S**	3,4	Y	Increased recording of alcohol consumption.	Y	Y	Y
Olfson 1992 USA Alcohol [74]	884	110	1	Clinical prompt: addition of CAGE to health form completed by first-time pts. prior to first consult.	**S**	4	N	Alcohol problem detection (either problem drinking or abuse) increased cf. baseline.	N	N	N
Seale 2015 USA Alcohol [75]	1318	na	4	Partial funding for coordinator; coordinator + clinician training; implementation committees; implementation guide + freedom to adapt to local setting; progress feedback.	**S, BI,** RP, RT	3,4	Y	Increased record of any screening or validated screening. Increased identification of risky users. Increased record of BI.	?	Y	?

^a^Author, year, country (as three-letter ISO 3166 country codes), focus and citation are given; ^b^significant positive result for clinical action is indicated in bold; Y – Yes, N – No; S – screening; BI – brief intervention; RP – relapse prevention medicines; PT – psychosocial therapies; RT – referral to treatment; Strategy targets: Clin. Action – clinical action, Org. level – organisational level (1 = National, 2 = Health System, 3 = Practice, 4 = Clinician, 5 = Patient), M/faceted – multifaceted; ^c^CQI elements: 1- Using ‘systematic data guided activities’ to identify problems and achieve improvement; 2 - ‘designing with local conditions in mind’ i.e. adapting and or designing strategies to fit the special characteristics of the local setting; 3 - using an ‘iterative development and testing process’; na – not available in article; (c) – control; (i) – intervention; pt. – patient; MR – medical record, eMR electronic medical record,.? – unclear; cf. – compared with

All studies were conducted in member countries of the Organization for Economic Co-operation and Development (OECD) and all countries but one were part of the Group of Twenty (G-20). Twenty-four reports represented projects conducted exclusively in the United States of America (USA), 12 in Australia, seven in the United Kingdom, seven in individual European countries and one in Canada. Three reports were from the Optimizing Delivery of Health Care Interventions (ODHIN) trial, which reported on aggregated outcomes in five European countries, and two reports from the international WHO Collaborative Project. The clinical setting was predominantly a generalist, general practitioner-led PHC service; however, four reports [28, 35, 52, 57] representing three projects were conducted in nurse-led community health centres. Likewise, populations served by these were general, except two in adolescent PHC and four in PHCs predominantly serving Indigenous peoples [54, 63, 65, 78].

### Targeted clinical actions

The majority of reports (52/56) examined improvement in rates of screening or brief intervention (BI) and/or referral to treatment. Twenty-four reports recommended or reported on the use of a validated screening measure, with 14 using either AUDIT (Alcohol Use Disorders Identification Test), its shorter version, AUDIT-C or both. Other validated screening tools included Single Alcohol Screening Question (SASQ), Fast Alcohol Screening Test (FAST), CAGE (an acronym for its four questions) and Short Michigan Alcohol Screening Test (sMAST). There was a wide range of terms used to describe screening and BI. For example, asking about alcohol consumption, eliciting alcohol history, ‘assessment’ of alcohol consumption or similar was used for screening; ‘brief advice’, ‘brief counselling’, and discussing alcohol was used for BI. Only six reports addressed improvement in rates of pharmacotherapy uptake for relapse prevention [31, 42, 43, 49, 61, 75]. None included implementation of psychosocial therapies, though referral to such therapies was mentioned as a treatment option.

### Characteristics of improvement strategies

#### Types and levels of implementation

A variety of strategies were employed to improve screening and treatment for unhealthy alcohol use. These targeted one or more different organizational levels. Of the 56 reports, none targeted all five levels, and only two were targeted at four levels. The majority of reports (50/56) included clinicians as targets, followed by the practice (35/56), with 32 reports targeting both. Of those, only nine reports also targeted the health-system level, and two reports targeted four levels, including the patient. Table 3 summarizes strategies by implementation level. Most implementation strategies (42/56) were multifaceted. Of these, 33 targeted two or more organizational levels.

**Table 3:** Types of strategy components employed by level of implementation

Organizational level	Strategy components	Reports
National	Pay-for-performance schemes, computer templates, grants for training initiatives	[50, 53]
Health system	Network meetings, audit and feedback, performance measures, changes to information systems, training, policy and leadership engagement, implementation committees, pay-for-performance schemes	[44, 48–50, 52, 53, 57, 59–62, 67, 70]
Practice	Training, telephone and on-site support, written and electronic materials, practice procedures and workflow changes, financial incentives, audit and feedback, involvement of staff other than clinicians, local champions and implementation committees, introduction of specialist staff, change to consultation booking time, systems audits and support in design of improvement strategies, information sharing between sites	[23–26, 28, 32, 33, 35, 36, 40, 41, 43, 44, 46, 49, 51, 52, 54–63, 65, 68–70, 73, 75, 77, 78]
Clinician	Training, telemarketing, letters to prescribers, academic detailing, written and electronic materials/guidelines, clinical prompts, audit and feedback, facilitation of referrals	[23–47, 49, 52, 54–57, 59–77]
Patient	Patient activation by: pre-appointment self-assessment +/−personalized feedback, information/resource mailouts	[37, 46, 49, 59]

#### Duration of implementation and follow-up

For studies where it was possible to extract these data, the median duration of the implementation phase was 28.2 weeks (IQR = 40, *n* = 50), and median duration from commencement of implementation to last data collection was 52 weeks (IQR = 52, *n* = 53). Of the 49 reports, where both types of duration data were available, 20 had their last data collection event after the end of implementation phase, indicating a follow-up period.

### Reports with CQI elements

Of the included reports, 22 described strategy components that were consistent with at least one of the three essential CQI elements (Table 4) [23]. An attempt to design or allow adaptation of implementation strategies to fit local conditions was the most commonly identified element (*n* = 20), followed by the use of iterative development and testing processes (*n* = 14). Using ‘systematic data guided activities’ to identify problems and achieve improvement, such as responding with corrective actions to regular practice audit reports and monitoring implemented changes,was identified in 13 reports.

**Table 4:** Distribution of CQI elements

Element (i) Data-guided	Element (ii) Local tailoring	Element (iii) Iterative process	Number of reports (*N* = 56)
–	–	–	34
–	–	Y	1
–	Y	–	6
–	Y	Y	1
Y	Y	–	1
Y	Y	Y	12

(i) Using ‘systematic data guided activities’ to identify problems and achieve improvement; (ii) ‘designing with local conditions in mind’ i.e. adapting and or designing strategies to fit the special characteristics of the local setting; (iii) using an ‘iterative development and testing process’

All three essential CQI elements were clearly identifiable in 12 reports. Of these, three were RCTs and seven were focused on broader prevention of risky behaviours (rather than being solely focused on unhealthy alcohol use). All examined screening and/or BI. Two also examined relapse prevention medicines. In contrast to other reports, more studies with all CQI elements targeted health system practice and clinician levels for implementation strategies and all were multifaceted (Table 5). Studies with all CQI elements also had higher median implementation duration.

**Table 5:** Key characteristics of reports with three CQI elements compared to other reports

Characteristic	Number of reports		
	Reports with 3 CQI elements (%), *n* = 12	Other reports (%), *n* = 44	All reports (%), n = 56
Multifaceted	12 (100)	30 (68.2)	42 (75.0)
Randomized	3 (30.0)	21 (45.7)	24 (42.9)
Alcohol-specific	5 (41.7)	30 (68.1)	35 (62.5)
Studied patient outcome	1 (8.3)	4 (9.1)	5 (8.9)
Included patient as target level	0 (0.0)	4 (9.1)	4 (7.1)
Health-system + practice + clinician as target level	4 (33.3)	5 (11.4)	9 (16.1)
Implementation duration (weeks)^a^:	(*n* = 11)	(*n* = 39)	(n = 50)
Median	52.0	21.7	28.2
Interquartile range	39	35.7	40
Implementation to end of data collection (weeks)^a,b^	(*n* = 11)	(*n* = 42)	(n = 53)
Median	104.0	38.3	52.0
Interquartile range	65	64.3	84

^a^Some data were missing due to lack of detail in reports

^b^this duration was defined as beginning of implementation until the last data collection event

#### Outcomes in relation to type of implementation strategy

The majority of reports (*n* = 51, 91.1%) showed a statistically significant increase in utilization of at least one clinical action. Significant increases in implementation were shown most often for screening and least often for referrals (Table 6). Only five reports (8.9%) included patient outcomes [29, 35, 40, 44, 45]. Of those, one [44] reported on changes in blood pressure and the rest on patient-reported changes in alcohol consumption. No significant between-group differences in these outcomes were shown, although there were some significant within-group outcomes in two reports [44, 45].

**Table 6:** Reports with significant positive implementation outcomes by clinical action

Clinical action	Reports with 3 CQI elements (*n* = 12)		Other reports (*n* = 44)	
	Examining action	Reporting increased utilization(% reports)	Examining action	Reporting increased utilization(% reports)
Screening	11	9 (81.8)	33	25 (75.6)
Brief Intervention	9	6 (66.7)	33	19 (57.6)
Pharmacotherapies	2	1 (50.0)	4	3 (75.0)
Referral	4	0 (0.0)	10	3 (30.0)

The proportion of reports with any positive outcome was similar in the 12 reports that included all three essential CQI elements, compared with the 44 reports that did not (91.7% compared to 90.1%). However, a higher proportion of the reports with three CQI elements achieved a significant improvement for two of the examined clinical actions: 81.8% for screening, 66.7% for brief intervention (compared with 75.6 and 57.6% respectively). Of the two reports with all CQI elements that aimed to examine pharmacotherapies, one reported a significant improvement and the other did not report results specific to this action. Of the five reports that presented patient outcomes, one [44] had all three CQI elements and reported a significant within-group improvement of systolic blood pressure but not between-groups.

## Discussion

This is the first systematic review to investigate incorporation of CQI elements into strategies to improve implementation of screening and treatment for unhealthy alcohol use in primary care. There was much variation in the studies’ design and delivery and studies concentrated mainly on screening and brief intervention for non-dependent alcohol use. There was little work on implementing onsite management of alcohol dependence, for example, pharmacotherapy for relapse prevention. Only 12 studies included all three CQI elements considered core to the CQI approach, while 22 studies incorporated at least one CQI element.

General practitioners are most often the first point of contact with healthcare for any drinkers. Therefore, it is important that PHCs are equipped to deal with the full spectrum of unhealthy alcohol use. Currently, screening and brief intervention are widely advocated as an effective secondary prevention approach for hazardous or harmful alcohol use in PHC settings [6]. If more severe alcohol problems are detected during the course of screening and brief intervention, referral to treatment away from the PHC service is often used. However, there is little evidence that this approach actually leads to effective linking with specialised services for patients who need them [79]. Furthermore, in many settings referral to specialist healthcare may not be an option due to costs, geographic isolation, long waiting periods or associated stigma. Thus, prescription of relapse prevention medicines in PHC rather than by referral to specialist centres may result in increased patient engagement at the point of detection or when the patient may be motivated and open to change.

Yet in the large volume of literature reviewed, only six studies included pharmacotherapies for relapse prevention as a target of implementation strategies. Only four of these also included BI for non-dependent (hazardous or harmful) drinkers, thus addressing the full range of unhealthy alcohol consumption.

### Types, levels and duration of strategies used to improve implementation

Strategies that are alcohol-specific, multifaceted and target multiple organizational levels have previously been shown to be associated with improved implementation outcomes [7, 8]. While the reviewed reports all tended to display some combination of these characteristics, reports with all three CQI elements more commonly utilized multifaceted designs and targeted the practice and health system levels (33.3%) than reports without these elements (11.4%). Overall, fewer studies incorporated the patient-level action as a target of implementation (none of the reports with three CQI elements and four of the other reports). This warrants more attention as there is evidence that strategies that include patient-oriented components of action (e.g. mailouts) in combination with other levels may be better at decreasing alcohol consumption than clinician-oriented strategies alone [8].

We found that details of study duration were often lacking in the included reports either due to omission or the nature of the study design. It was often difficult to distinguish the duration of individual phases of the study: baseline, implementation and follow-up, making systematic data extraction challenging. We therefore used the duration from start of strategy implementation to end of data collection as a proxy for study duration. When the end of data collection was later than duration of implementation, this was considered as an indication of follow-up. The median study duration for studies with all three CQI elements was much higher than for studies without these (104 and 38.2 weeks respectively). In addition, only 20 reports had a clear indication of follow-up after the conclusion of active implementation. While there is evidence that study duration of 12 months or more is a significant predictor of improvement in BI implementation [8], it is not clear whether this is due to longer duration of implementation or longer follow-up. The lack of consistent duration data on implementation and follow-up is an important gap in the evidence-base as these are likely to influence the uptake of the implementation strategy, its sustainability and effects on both service-level and patient outcomes. For example, longer duration of implementation may be necessary to implement more complex treatment regimens and to allow for late adopters. Insufficient duration and frequency of follow-up may also lead to loss of information about potential improvements in patient outcomes as well as optimal length of strategy implementation to ensure sustainability.

While studies with all three CQI elements appear to have more favourable design characteristics than studies without these, it is less clear if this leads to better outcomes in improving delivery of screening and treatment for unhealthy alcohol use. A higher proportion of reports with three CQI elements improved screening outcomes and, to a lesser extent BI outcomes. However, they did not improve uptake of pharmacotherapies. These results need to be interpreted with caution, given the small number of reports with all three CQI elements, and even smaller number (*n* = 2) of these that investigated use of pharmacotherapies.

It is notable that three of the four reports on studies in indigenous settings included all essential CQI elements. These represented two CQI studies (in Australia and US), both set in community controlled health services. This perhaps reflects CQI’s suitability to facilitate efficient service improvements in settings where lack of adequate resources and multiple health priorities can be a challenge and where stakeholder-driven, culturally relevant programs are crucial [80]. Finally, very few implementation studies (*n* = 5) reported patient outcomes and those that did were unable to demonstrate significant reductions in patient alcohol consumption [8]. Just one report considered patient outcomes other than alcohol consumption. The demonstrated lack of evidence of significant effect on patient outcomes may be due to not enough consideration being given to the complexity of studies that test implementation strategies as well as effectiveness of clinical interventions [81, 82].

### Recommendations for practice and research

#### Improving screening and treatment uptake in PHC

Given the dearth in evidence, there is a need for more implementation studies on treatment for the full spectrum of unhealthy alcohol use, particularly the use of pharmacotherapies to treat dependent drinkers. This is particularly important in low-income countries where alcohol-attributable mortality is highest [83], where specialist services may be limited, but where few such studies are conducted.

The effectiveness of implementation strategies may depend on how well they fit the services’ own circumstances, address the barriers to implementation and how they can co-exist with existing local enablers in a specific service. Furthermore, studies rarely analyse the contribution of individual components of the studied strategies to the overall effect on service-level outcomes. This detail could help services tailor their approaches to improving screening and treatment for unhealthy alcohol use. The fact that uptake of screening and treatment for unhealthy alcohol use in PHC remains low [84] suggests that future research effort should concentrate on “service-friendly” strategies as they may increase uptake and sustainability of effect.

The CQI approach provides a framework for how to carry out an improvement process systematically and on an ongoing basis. What activity is carried out to achieve the improvement and how it is measured is left up to the services to decide. Services can work towards a national benchmark or choose their own implementation goal. If implemented well, the CQI approach can offer the advantage of being sensitive and responsive to local conditions, and to newly arising challenges. The Plan-Do-Study-Act of the CQI cycle can facilitate the identification of the optimal combination of strategy components for a particular clinical setting. It is compatible with reflective learning and change to enable interventions to adapt to complex environments [85]. However, there may be barriers to implementation of CQI itself, including staff time and resources [86].

#### CQI in implementation research

The ultimate goal of implementing and improving service delivery is to improve patients’ health, but evidence for this in relation to screening and treatment of unhealthy alcohol use is lacking. One approach to closing this evidence gap could be to simultaneously test implementation strategies and the effectiveness of clinical interventions through hybrid designs. This approach is thought to enable a more rapid generation of evidence base for the clinical interventions in “real life” settings than the traditional stepped processes: efficacy-effectiveness-implementation [81, 82].

Implementation research utilizing hybrid designs and quality improvement research can complement each other, with the former contributing more rigorous, scientifically robust summative evaluation and the latter providing information to enable a systematic refinement of the studied implementation strategy [87]. Inclusion of CQI in implementation research, particularly in hybrid designs thus has the potential to provide the optimal study design: flexible and responsive implementation strategies, scientific rigor to detect improvements in both service and patient-level outcomes, and ability to simultaneously provide information of value to healthcare managers and policy makers.

### Limitations

Due to the volume of work and logistic constraints the search was limited to English language and only peer-reviewed literature was included in this study. Some health organization-based programs are published only in annual and commissioned reports and so would not have been included. However, a review of grey literature was out of scope of this review.

It has been previously noted that CQI studies are not easily identifiable in the academic literature as these are often not reported clearly or consistently [15, 22, 88]. Furthermore, the word and formatting limits of peer reviewed journals may contribute to underreporting and imprecise reporting of CQI methods [15]. Data extraction in this review was done by single person (MD) because of resource constraints. This may have introduced bias to the coding of key characteristics of strategies, particularly the three CQI elements. However, a priori definitions and clear criteria were used to reduce subjectivity.

Future meta-analysis of these studies may offer deeper insights into the benefits of incorporating elements of CQI into implementation research in alcohol service delivery. However, the heterogeneity of the studies, gaps in reporting and generally low numbers of reports that meet the inclusion criteria will pose challenges.

## Conclusions

The uptake of screening and treatment of unhealthy alcohol use in PHC continues to be low despite national and international guideline recommendations. Many studies of implementation strategies have yet to show significant improvement in patient outcomes. There remains a lack of implementation studies for treatment for the full spectrum of unhealthy alcohol use. There is also a lack of information in the effectiveness of particular components of multifaceted strategies, or inclusion of patient-level implementation strategies and outcomes. Incorporating CQI elements into implementation strategies may offer promise as an approach to deliver flexible and responsive solutions for sustained implementation of alcohol care. However, further well-designed research, including hybrid studies of both implementation and patient outcomes are needed to draw clearer conclusions on the most effective way to implement screening and treatment for unhealthy alcohol use in PHC.

## Data Availability

The search strategy used to generate the initial systematic review search result is available in PROSPERO (protocol ID CRD42018110475). https://www.crd.york.ac.uk/prospero/. The datasets used and/or analysed during the current study are available from the corresponding author on reasonable request.

## Abbreviations

AUDIT: Alcohol Use Disorders Identification Test
BI: Brief intervention
CAGE: Alcohol screening tool; abbreviation in an acronym for its four questions
CQI: Continuous Quality Improvement
EPOC: Effective Practice and Organisation of Care
FAST: Fast Alcohol Screening Test
G-20: Group of Twenty
ICD11: International Classification of Diseases, 11th Revision
MeSH: Medical Subject Headings
ODHIN: Optimizing Delivery of Health Care Interventions
OECD: Organization for Economic Co-operation and Development
PHC: Primary health care
RCT: Randomized controlled trial
SASQ: Single Alcohol Screening Question
SBI: Screening and brief intervention
sMAST: Short Michigan Alcohol Screening Test
WHO: World Health Organization
